# Dynamics of a Novel Highly Repetitive CACTA Family in Common Bean (*Phaseolus vulgaris*)

**DOI:** 10.1534/g3.116.028761

**Published:** 2016-05-16

**Authors:** Dongying Gao, Dongyan Zhao, Brian Abernathy, Aiko Iwata-Otsubo, Alfredo Herrera-Estrella, Ning Jiang, Scott A. Jackson

**Affiliations:** * Center for Applied Genetic Technologies, University of Georgia, Athens, Georgia 30602; † Department of Horticulture, Michigan State University, East Lansing, Michigan 48824; ‡ Laboratorio Nacional de Genómica para la Biodiversidad, Cinvestav Sede Irapuato, Mexico 36821

**Keywords:** DNA transposon, CACTA, genome evolution, common bean, *Phaseolus*

## Abstract

Transposons are ubiquitous genomic components that play pivotal roles in plant gene and genome evolution. We analyzed two genome sequences of common bean (*Phaseolus vulgaris*) and identified a new CACTA transposon family named pvCACTA1. The family is extremely abundant, as more than 12,000 pvCACTA1 elements were found. To our knowledge, this is the most abundant CACTA family reported thus far. The computational and fluorescence *in situ* hybridization (FISH) analyses indicated that the pvCACTA1 elements were concentrated in terminal regions of chromosomes and frequently generated AT-rich 3 bp target site duplications (TSD, WWW, W is A or T). Comparative analysis of the common bean genomes from two domesticated genetic pools revealed that new insertions or excisions of pvCACTA1 elements occurred after the divergence of the two common beans, and some of the polymorphic elements likely resulted in variation in gene sequences. pvCACTA1 elements were detected in related species but not outside the *Phaseolus* genus. We calculated the molecular evolutionary rate of pvCACTA1 transposons using orthologous elements that indicated that most transposition events likely occurred before the divergence of the two gene pools. These results reveal unique features and evolution of this new transposon family in the common bean genome.

Transposable elements (TEs) are ubiquitous DNA sequences that can change their positions within a genome or transfer horizontally among genomes (Gilbert *et al.* 2010). TE movements may cause mutations, affect gene expression, and change genome sizes and structures; therefore, they are considered an important force in gene and genome evolution (Kazazian 2004). TEs are grouped into two major classes according to their transposition mechanism and sequence features. Class I elements, retrotransposons, mobilize via a copy-and-paste model and have the potential to dramatically increase copy number; whereas, class II elements, DNA transposons, transpose via a cut-and-paste model or rolling-circle replication (Kapitonov and Jurka 2001; Wicker *et al.* 2007). Class II elements have been further divided into 12 superfamilies (Feschotte and Pritham 2007; Wicker *et al.* 2007). Except for the Helitron and Crypton superfamilies, all class II elements have terminal inverted repeats (TIRs). To date, only six superfamilies of DNA transposons have been described in plants, including *Tc1/Mariner*, *hAT*, *Mutator*, *PIF/Harbinger*, CACTA, and *Helitron*; other DNA transposons have been identified in either animals or microbes (Feschotte and Pritham 2007; Wicker *et al.* 2007). Each transposon class contains both autonomous and nonautonomous elements; the former encode protein(s) necessary for transposition (*e.g.*, transposases for class II elements), whereas the latter do not possess functional transposases and their movement is catalyzed by their autonomous partners.

Superfamily CACTA transposons were first identified in maize and named *Enhancer* (*En*) and *Suppressor - mutator* (*Spm*) (Peterson 1953; McClintock 1954), and have since been found in many plants and invertebrates (Vodkin *et al.* 1983; Shirsat 1988; Chopra *et al.* 1999; Kawasaki and Nitasaka 2004; Feschotte and Pritham 2007). Members of this superfamily exhibit a common terminal sequence (5′-CACTA-3′), the length of their TIRs vary from 10–54 bp, and generate a 3 bp target site duplication (TSD) upon insertion (Kunze and Weil 2002; Feschotte and Pritham 2007). Both autonomous and nonautonomous CACTA transposons have been identified, and some elements are more than 20 kb in size (Kunze and Weil 2002; Zabala and Vodkin 2008). In addition to their use for insertional mutagenesis in plants (Wisman *et al.* 1998; Kumar *et al.* 2005), CACTA transposons have been reported to carry host gene sequences (Kawasaki and Nitasaka 2004; Zabala and Vodkin 2007), thus they provide a way to create new functional genes by capturing and rearranging genic fragments, an important mechanism for gene and genome evolution (Zabala and Vodkin 2007).

Legumes represent the third largest family of flowering plants and the second most economically and agriculturally important plant family to humans after Grasses (Poaceae), they also have unique roles in nitrogen fixation and natural ecosystems (Graham and Vance 2003). To date, ten legume genome sequences have been published, including *Lotus japonicas* (Sato *et al.* 2008), soybean (Schmutz *et al.* 2010), pigeon pea (*Cajanus cajan*) (Varshney *et al.* 2011), alfalfa (*Medicago sativa*) (Young *et al.* 2011), chickpea (*Cicer arietinum*) (Varshney *et al.* 2013), common bean (Schmutz *et al.* 2014; Vlasova *et al.* 2016), mungbean (*Vigna radiata*) (Kang *et al.* 2014) and two wild peanuts (Bertioli *et al.* 2016). These resources make the legumes an excellent system to study aspects of domestication, polyploidization, and genome evolution using comparative genomics.

The *Phaseolus* genus belongs to the legume (Fabaceae) family and contains 45 wild and five domesticated species, including runner bean (*P. coccineus*), tepary bean (*P. acutifolius*), lima bean (*P. lunatus*), year bean (*P. dumosus*), and common bean (*P. vulgaris*) (Delgado-Salinas *et al.* 1999). It was estimated that members of the *Phaseolus* genus radiated less than 6 million years ago (MYA) and shared common ancestors with cowpea (*Vigna unguiculata*) and soybean (*Glycine max*) around 8 and 20 MYA, respectively (Supplemental Material, Figure S1) (Lavin *et al.* 2005; Delgado-Salinas *et al.* 2006). Common bean is the most economically important species of the genus *Phaseolus* and is grown and consumed worldwide. In some developing countries, it is a major source of calories and protein and an important source of income for small-holder farmers (Blair *et al.* 2011). Common bean is native to tropical America and previous studies have shown that cultivated common bean was domesticated from two diverged wild gene pools, Andean and Mesoamerican, which diverged from a common ancestor ∼111,000 years ago (Velasquez and Gepts 1994; Mamidi *et al.* 2013). The two gene pools, represented by both wild and cultivated forms, can be distinguished at the morphological and molecular levels and exhibit incomplete reproductive isolation (Gepts 1998).

Here, we analyzed genome sequences from common bean varieties, G19833 and BAT93, which represent two domesticated gene pools of common bean, Andean and Mesoamerican, respectively. We identified a new CACTA transposon family named pvCACTA1 that was extremely abundant in both genomes and appears to be restricted to the *Phaseolus* genus. We found that the pvCACTA1 transposons preferentially insert into AT-rich regions. We compared the distributions of pvCACTA1 between the two common bean genomes and identified polymorphisms, some of which were in genic regions. Sequence divergence of pvCACTA1 elements suggested that massive transpositions likely occurred before the split of the two gene pools. These results indicate that the pvCACTA1 transposons played important roles in genomic divergence of common bean, though how this contributed to morphological diversification and/or local adaptation remains to be explored.

## Materials and Methods

### Plant materials

A total of 33 plant genotypes are used in this study, including seven cultivated common beans from the Andean gene pool (G19833, G04703, G12408, G21063, G05828, G04627, and G00111), five cultivated common beans from the Mesoamerican gene pool (BAT93, G01795A, G22078, G17648 and G03825), five Andean wild accessions (G23580, G19891, G07273, G02563, and G12578), seven Mesoamerican wild accessions (PI535416, G10999, G11051, G12910, G13505, G21113, and G19907), seven cultivated and wild species from the *Phaseolus* genus (*P. coccineus*, *P. lunatus*, *P. acutifolius*, *P. dumosus*, *P. leptostachyus*, *P. hintonii*, and *P. maculatus*), soybean (Williams 82) and cowpea. All these plants were grown in the greenhouse at the University of Georgia and the young leaves were used to extract DNAs.

### Sequence data sources

The genome sequences of two common bean genotypes, G19833 and BAT93, were obtained from the Phytozome website (www.phytozome.org) and the PhasIbeAm team website (http://mazorka.langebio.cinvestav.mx/phaseolus), respectively. Thirteen common bean BAC sequences, including three BACs from BAT93, FJ817289–FJ817291 (David *et al.* 2009), and 10 BACs from G19833, GU215957–GU215966 (Lin *et al.* 2010), were downloaded from GenBank. The gene sequences in G19833 annotated by the common bean genome sequence project (Schmutz *et al.* 2014) were used to detect the transposon-related genes. The annotated genes and Gene Ontologies in *Arabidopsis thaliana* were downloaded from the *Arabidopsis* Information Resource website (http://www.arabidopsis.org).

### Sequence analysis

To identify transposons in common bean, 10 BACs from G19833 were used to conduct all-against-all BLASTN searches. All putative repetitive sequences were manually inspected and to define complete transposons based on the terminal sequences and TSDs. The identified small transposons were used as a library to screen the common bean genome sequences with Repeatmasker (http://www.repeatmasker.org). The program was run using the default parameters but ‘nolow’ option. In addition, we also set a cut-off score of > 250, and a hit sequence length of > 50 bp. Any read matching the criteria was considered as a potential transposable element. Complete CACTA elements were determined by TIRs and 3 bp TSDs; 200 bp flanking sequences from each side and 3 bp TSDs were extracted and used to calculate the nucleotide frequency of the transposon insertion sites. To generate the sequence logo, the 20 bp immediately flanking sequences (10 bp for each side) of all complete elements and their 3 bp TSDs were extracted and aligned with the Seq2Logo program (http://www.cbs.dtu.dk/biotools/Seq2Logo).

### Transposon display

The transposon display was performed following the previous study (Casa *et al.* 2000). Briefly, 500 ng genomic DNA was digested with *Mse*I (New England, Ipswich, MA) at 37° for 3 hr. For double strand adapter preparation, the mixture from a half of 100 mM adapter forward primer (5′-GACGATGAGTCCTGAG-3′) and a half of 100 mM adapter reverse primer (5′-TACTCAGGACTCAT-3′) was mixed and denatured at 95° for 5 min, then annealed at room temperature for 2 hr. The digested genomic DNA and the adapters were ligated using a T4 DNA ligase kit (New England Biolabs, Ipswich, MA,) at 4° overnight. Preselective amplification was carried out with transposon-specific primer (5′-CTCTAATTTAGTTAATATTGCA-3′) and adapter primer (5′-GACGATGAGTCCTGAGTAG-3′). The PCR reaction consisted of 1 × PCR buffer, 0.2 mM dNTP, 1.5 mM MgCl_2_, 0.2 μM of each primer, 2 μl diluted (1:4 with deionized water) digested DNA with adapter, and 1.0 unit of Taq polymerase (Invitrogen, Carlsbad, CA) in a total volume of 20 μl. The reaction was denatured at 94° for 3 min, then continued with 30 cycles of 45 sec of 94° denaturation, 45 sec of 53° annealing, and 45 sec of 72° extension, and a final cycle at 72° for 5 min to terminate the reaction. PCR for selective amplification was conducted using the transposon primer (5′-GCAACTAATTATTTTGGTTTCTA-3′) and adapter primer (5′-GACGATGAGTCCTGAGTAGA-3′). 5 μl PCR products were examined by electrophoresis on a 1% agarose gel. The remaining 15 μl PCR product was diluted 10 times with 0.1 × TE buffer.

The reaction consisted of 1 × PCR buffer, 0.2 mM dNTP, 1.5 mM MgCl_2_, 0.25 μM of adapter primer, 0.25 μM transposon primer for selective amplification, 1 μl diluted preselective PCR product, and 1 unit of Taq DNA polymerase (Invitrogen, Carlsbad, CA) in a total volume of 25 μl. PCR conditions were as follows: 94° 3 min; 94° 45 sec, 63° 45 sec (−1° per cycle), 72° 45 sec for 6 cycles; 94° 45 sec, 56° 45 sec, 72° 45 sec for 30 cycles; a final cycle of 72° for 5 min. The PCR reaction was mixed with 20 µl of formamide loading buffer and denatured at 95° for 5 min, then placed on ice for 5 min. 6 μl of the mixture was loaded on a 6% denaturing polyacrylamide gel and run for 2 hr at a constant 1500 V. The gel was silver-stained following Promega’s silver sequence DNA sequencing system (Promega, Madison, WI).

### FISH analysis of transposon

The fresh roots of common bean were collected and fixed in 3:1 ethanol and glacial acetic acid for 24 hr at room temperature, and then stored at 4°. Mitotic chromosomes were prepared following published protocols (Findley *et al.* 2010). FISH was carried out according to our previous protocol (Walling *et al.* 2005). Slides were stored at −80° until use. The DNA of common bean was amplified with the primers (Forward, 5′-CCAATTTTTAGAGACCAAAAT-3′; Reverse, 5′-TTGCATACATTCTTGCTTTTCA-3′) and the PCR product covering ∼300 bp pvCACTA1 sequence was used as the probe for FISH. The probe was labeled with digoxigenin dUTP (Roche Diagnostics, Indianapolis, IN), and visualized with anti-digoxigenin-rhodamine (Roche Diagnostics, Indianapolis, IN). The images were taken with Zeiss Axio Imager M2 microscope, equipped with AxioCam MRm, controlled by Axio Vision 40 V4.8.2.0. The images were adjusted for publication using Adobe Photoshop CS5.1 (Adobe Systems Incorporated).

### PCR analysis

To validate insertion polymorphisms of pvCACTA1 transposons, PCR analysis was conducted in a 25 μl amplification volume consisting of 20 ng genomic DNA, 1.5 mM MgCl_2_, 1.0 unit Taq DNA polymerase (Promega, Madison, WI), 0.2 mM dNTP, 0.2 mM primer, and 1 × PCR buffer. The temperature cycling conditions were 5 min at 95°, followed by 35 cycles of 95° for 45 sec; 55° for 50 sec; and 72° for 55 sec, and a final extension at 72° for 5 min. The amplification products were run on 1% agarose gels and stained with ethidium bromide. Four pairs of PCR primers were used including P1 (Forward: 5′-CGTGACAAAAAGCACAAGAAAG-3′, Reverse: 5′-TCAAGAGCCACAGAAGAAATCA-3′), P2 (Forward: 5′-GATGACCTGTCACCTAAAATG-3′, Reverse: 5′-AATACTCCTTCCTTCGTTTTT-3′), P3 (5′-CTTGCAAATTCAACTCCCAAAT-3′, Reverse: 5′-TGGTGGCCAATGTAAATAATCA-3′), and P4 (5′-ATTACATTCCACAATGCACCAA-3′, Reverse: 5′-AGGTGAATTTGGCCCTCTTTAT-3′). The target sites of four primer pairs are shown in Figure S4.

### Determining polymorphic transposons and subgrouping pvCACTA1 family

To detect polymorphic transposons, all complete pvCACTA1 elements and the 200 bp flanking sequences (100 bp for each side) were extracted and used as queries to search against the G19833 or BAT83 genomes using BLASTN. Polymorphic transposons were defined based on three criteria: 1) homologous sequences should be found for flanking regions on both sides of the element; 2) the flanking regions should be adjoined together in the reciprocal genome; and 3) no pvCACTA1 sequence within and between the flanking regions in the genome where absent. To avoid misalignment of flanking sequences, only the elements that inserted into regions with one hit were considered. Those located in repetitive sequences were discarded.

To subgroup the pvCACTA1 sequences, all complete elements in G19833 and BAT93 were used for all by all BLASTN searches and the output files were screened and summarized by a custom Perl script. The basic idea from grouping the elements is multiple rounds of comparisons. Each complete element was used to compare with other sequences, and any element that showed more than 85% sequence identity with the query sequence over more than 85% of its element size was grouped into the same subfamily. Additionally, sequences that were been grouped would not be used in subsequent rounds. The rank of subfamilies was sorted by the number of elements in the subfamily. For any subfamily, the query sequence served as the representative element to build the phylogenetic tree.

### Phylogenetic analysis

The complete pvCACTA1 transposons were aligned with the CLUSTALW program (Higgins *et al.* 1996) using the default options. The multiple sequence alignments were used to construct a phylogenetic tree using the neighbor-joining method in the MEGA 4 software (Tamura *et al.* 2007). The analysis was based on 1000 bootstrap replicates, using the nucleotide: maximum composite likelihood model.

### Data availability

The authors state that all data necessary for confirming the conclusions presented in the article are represented fully within the article.

## Results

### Discovery of a new and abundant transposon in common bean

**Figure 1:**
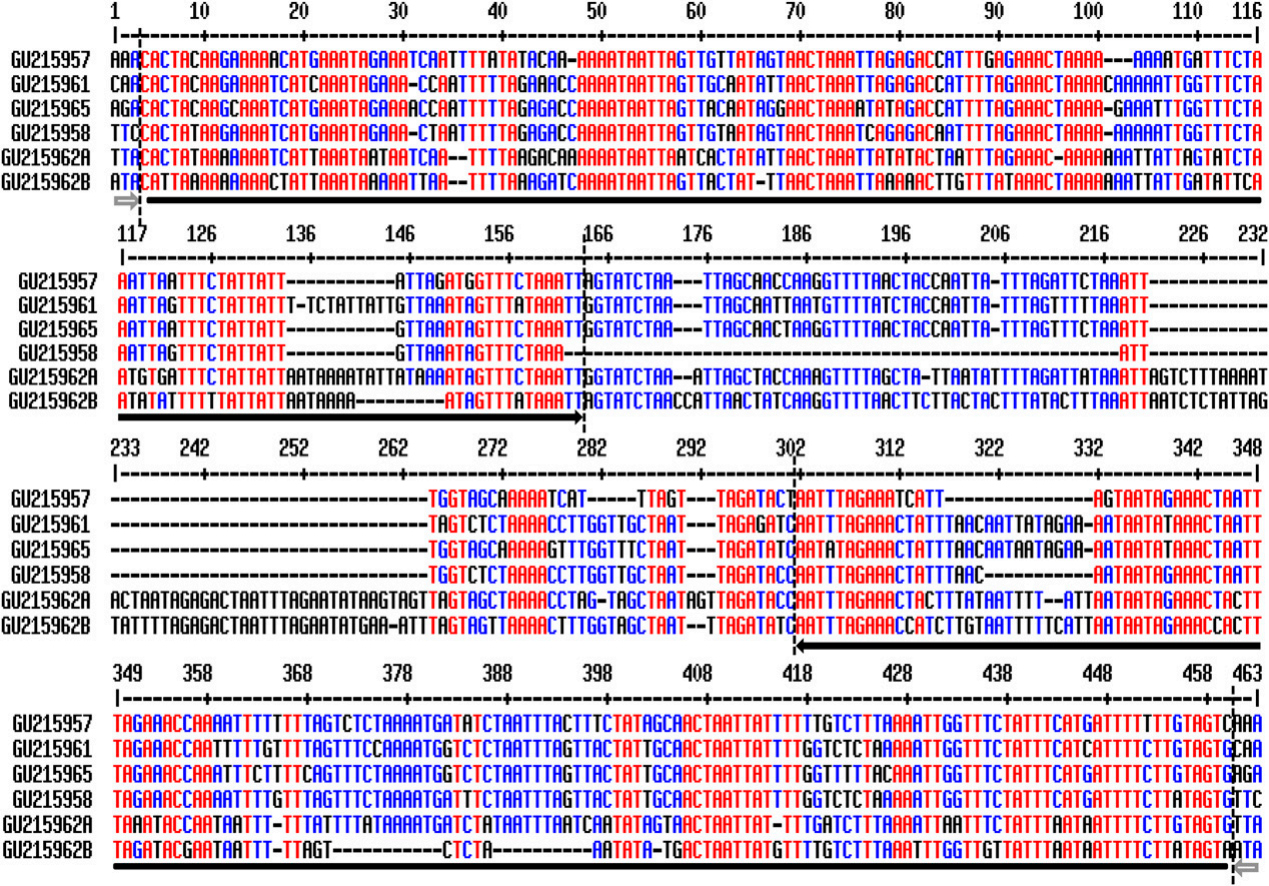
Sequence alignment of six pvCACTA1 complete elements identified in GU216957-215962. Black and gray arrows indicate TIRs and TSDs of the six transposons, respectively. TIRs, terminal inverted repeats; TSDs, target site duplications.

We previously generated ∼1 Mb genome sequence from common bean (Andean G19833; GenBank accession no. GU215957-GU215966), which was used for comparison to orthologous regions from soybean, to investigate the evolutionary fate of duplicated genes (Lin *et al.* 2010). We computationally analyzed these common bean sequences and found 24 transposons, including 18 complete LTR retrotransposons and six small DNA transposons. The six small transposons ranged from 330–446 bp and shared 67–88% sequence identity with each other. These sequences were further used as queries to search GenBank and available transposon databases including Repbase (http://www.girinst.org/repbase), plant repeat databases (http://plantrepeats.plantbiology.msu.edu), and the soybean transposon database (http://soybase.org/soytedb). No sequence identity was found between these elements and any known transposons, suggesting that the six elements were likely members of a new transposon family. Multiple sequence alignments revealed that all these elements were flanked by 3 bp TSDs and have TIRs with the typical motif of the CACTA superfamily (5′-CACTA…TAGTG-3′) (Figure 1), thus the transposons were considered to be a member of the CACTA family and we named it pvCACTA1. Unlike previous CACTA elements with short TIRs (Kunze and Weil 2002; Feschotte and Pritham 2007), members of the pvCACTA1 family had long TIRs ranging from 145–158 bp, even though the elements were small.

To find all elements of this transposon family in the common bean genome, the six pvCACTA1 sequences were combined with other identified TEs (Gao *et al.* 2014) and used as a TE library to screen the genome sequences of G19833 and BAT93 using RepeatMasker (www.repeatmasker.org). A total of 13,744 pvCACTA1 transposons were found in G19833, including 2523 complete elements as defined by the presence of both TIRs and TSDs. These elements covered 3.84 Mb, or more than 0.8% of the G19833 genome. This indicated that pvCACTA1 transposons were very abundant in G19833. To our knowledge, this may be the most abundant (high copy number) family among all CACTA transposons reported so far. A total of 13,118 pvCACTA1 elements were found in the BAT93 genome, including 2150 complete elements, accounting for 0.7% of the genome. The pvCACTA1 transposons were small and do not encode any protein sequence, thus they are nonautonomous DNA transposons, and their mobility depends on transposases produced by other transposons. However, we were unable to find any large autonomous transposon with sequence identity to the pvCACTA1 transposons.

The six elements were then used to conduct BLASTN searches against GenBank to identify homologous elements in other genomes. Five expressed sequence tags (ESTs) in runner bean were found that shared sequence identity with the pvCACTA1 elements, the most significant hit (GenBank accession no. CA916517) showed 71% sequence identity over a 396 bp region. Additionally, one EST (HO790818) in tepary bean showed significant sequence identity (*e* values of 7e^-25^) with the pvCACTA1 elements. No hits were found in any non-*Phaseolus* genomes, including published legume genomes such as *L. japonicas*, soybean, pigeon pea, alfalfa, and chickpea.

**Figure 2:**
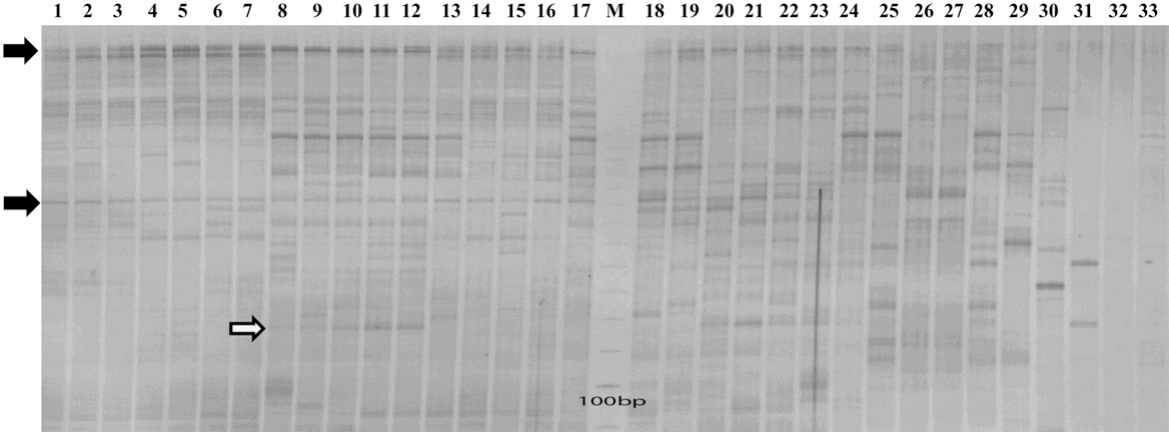
Transposon-display of pvCACTA1 family with primer targeted transposon TIRs and a *Mse*I adaptor primer. Lanes 1–24 are common bean: lanes 1–7 are seven cultivated Andean accessions (G19833, G04703, G12408, G21063, G05828, G04627, and G00111), lanes 8–12 represent five Mesoamerica cultivated accessions (BAT93, G01795A, G22078, G17648, and G03825), lanes 13–17 represent five wild Andean accessions (G23580, G19891, G07273, G02563, and G12578), lanes 18–24 indicate seven wild Mesoamerica accessions (G23582, G21245, PI535416, G10999, G11051, G12910, and G13505), and lanes 25–31 are seven *Phaseolus* species (*P. coccineus*, *P. lunatus*, *P. acutifolius*, *P. dumosus*, *P. leptostachyus*, *P. hintonii*, and *P .maculatus*), lane 32 and 33 mean soybean (*G. max*, Williams82) and cowpea (*V. unguiculata*), respectively. M is a 50 bp marker. The arrows filled with black and white denote bands shared by the two gene pools and a band specific to the Mesoamerican accessions, respectively. TIRs, terminal inverted repeats.

We further performed transposon display with a pvCACTA1-specific primer and the adaptor primer targeted to nearby cleaved restriction sites of *Mse*I enzyme to detect the distribution and polymorphism of pvCACTA1 elements. Amplification products were found in both cultivated and wild common beans, consistent with our computational analysis, and further confirmed the abundance of this transposon family in common bean as multiple bands were found. Amplification bands were also detected in seven other *Phaseolus* species including the four cultivated beans (runner bean, tepary bean, lima bean, and spotted bean), and three wild species, *P. dumosus*, *P. leptostachyus*, and *P. hintonii*. However, no product was found in either cowpea or soybean (Figure 2). We also conducted Southern blot analysis using the pvCACTA1 elements as a probe and no hybridization signal was detected in cowpea, soybean, pigeon pea, or alfalfa (data not shown). These results indicate that the pvCACTA1 transposons may be restricted to the *Phaseolus* genus. We found that some bands were shared by cultivated and wild accessions in both gene pools, indicating that these copies were inserted into the ancestral genome of common bean before the divergence of two gene pools. However, differences in amplification patterns were found between the two gene pools and seven *Phaseolus* species, revealing insertions/excisions of pvCACTA1 that occurred during speciation within the *Phaseolus* genus or that the targeted sequences have accumulated mutations. No band was amplified in soybean and cowpea, indicating that either there are homologous transposons of pvCACTA1 in these genomes but that they are diverged and cannot be targeted by these primers or that pvCACTA1 elements emerged after the divergence of the *Phaseolus* genus.

### Uneven distribution of pvCACTA1 in G19833

**Figure 3:**
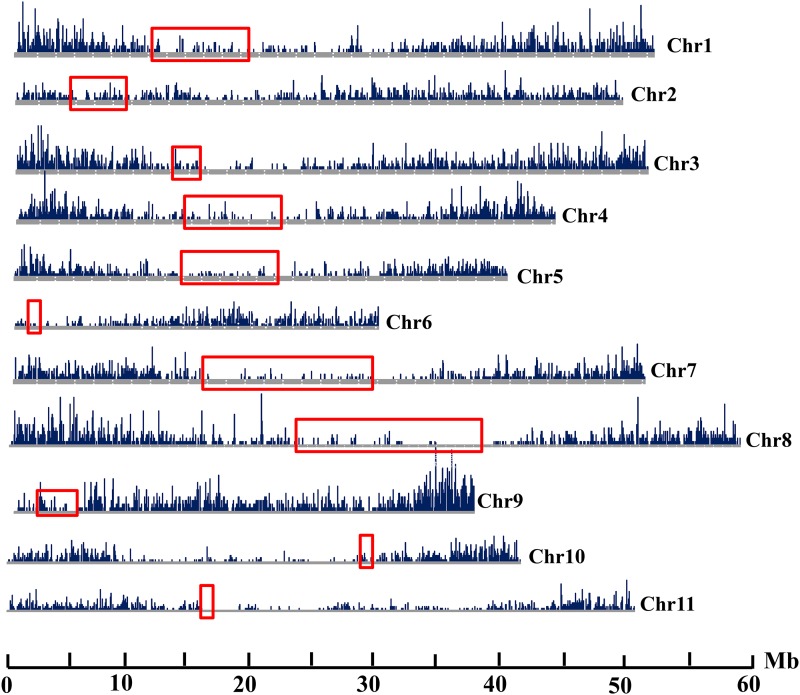
The genomic distribution of pvCACTA1 transposons in G19833. Red boxes indicate centromeres/pericentromeres. The unit of the y-axis was the number of pvCACTA1 transposons in a 100 kb window. Chr, chromosome.

pvCACTA1 elements were dispersed across the G19833 genome. However, the density varied among the 11 chromosomes and in different regions of each chromosome (Figure 3). The average transposon density was 29 pvCACTA1 elements per Mb sequence across the entire genome, but the 11 chromosomes had distinct transposon densities. Chromosome 9 had the highest element density (41.0 transposons/Mb), whereas chromosome 11 had the lowest (25.5 transposons/Mb). Additionally, pvCACTA1 transposons were distributed unevenly within chromosomes. The centromeric regions (red boxes in Figure 3), the boundaries of which were defined by the centromere-specific tandem repeats (Iwata *et al.* 2013), had significantly lower transposon densities than other locations (T test, *P* value = 8.4e^−08^). For example, transposon density in centromere 4 (*Cen4*) was one fifth of that in noncentromeric regions of chromosome 4. Additionally, some regions that surrounded the centromeres, such as the 21 Mb region (from 10–31 Mb) on chromosome 9, harbored a lower density of pvCACTA1 transposons; these likely represent the pericentromeric regions with lower recombination rates and lower gene densities (Schmutz *et al.* 2014). Thus, the pvCACTA1 elements were distributed unevenly across the common bean genome and some regions, particularly chromosome termini, have accumulated/retained more elements than other regions. These distribution patterns are different from many LTR retrotransposons in common bean and soybean which show higher density in pericentromeric regions (Schmutz *et al.* 2010; Gao *et al.* 2014).

**Figure 4:**
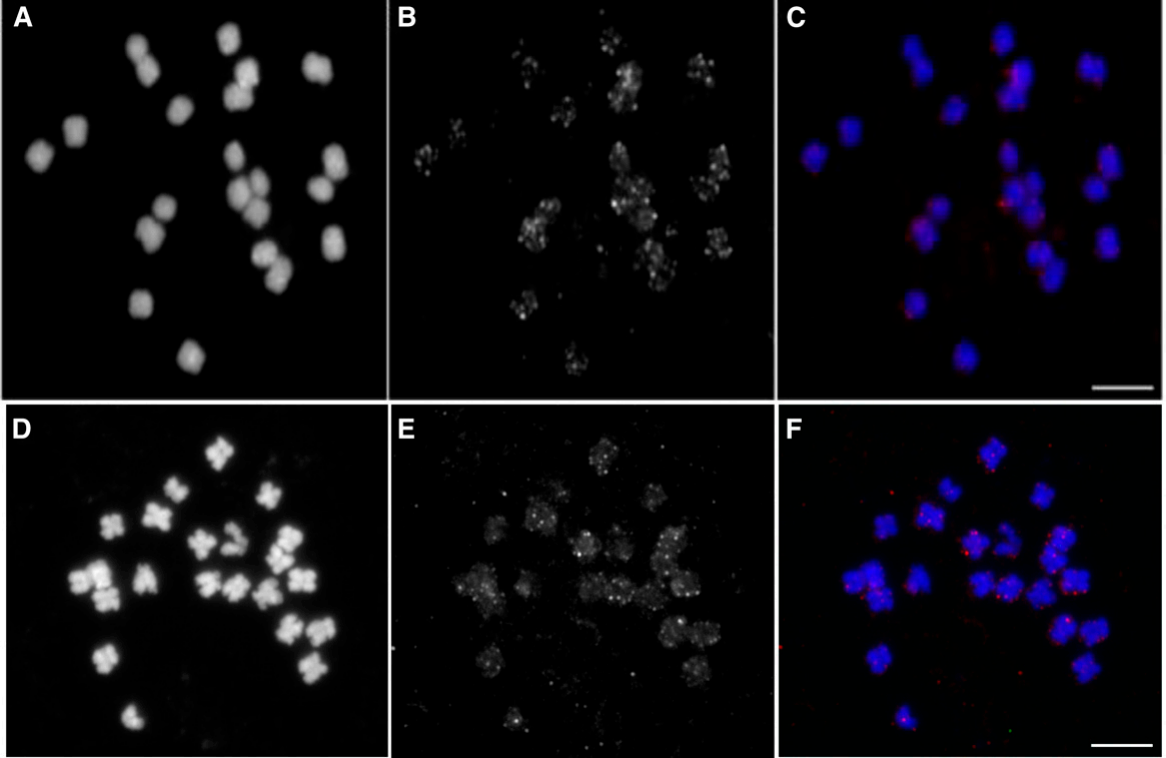
FISH of pvCACTA1 transposons in G19833 (A–C) and BAT93 (D–F). (A and D) Mitotic metaphase chromosomes counterstained with 4’, 6-diamidino-2-phenylindole (DAPI). (B and E) pvCACTA1 transposons. (C and F) Merged images. Scale bar = 5 µm. FISH, fluorescence *in situ* hybridization.

To further illuminate the chromosome distribution of pvCACTA1 transposons in common bean, we conducted FISH using pvCACTA1 elements as the probe. Hybridization signals were detected on all 22 mitotic chromosomes and the telomeres showed stronger signals than other regions (Figure 4). This result was consistent with our computational analyses and confirmed that the pvCACTA1 transposons were more abundant in telomeric regions in the G19833 genome. FISH results also revealed that the genomic distribution of pvCACTA1 transposons was similar between G19833 and BAT93.

### AT insertion site bias

**Figure 5:**
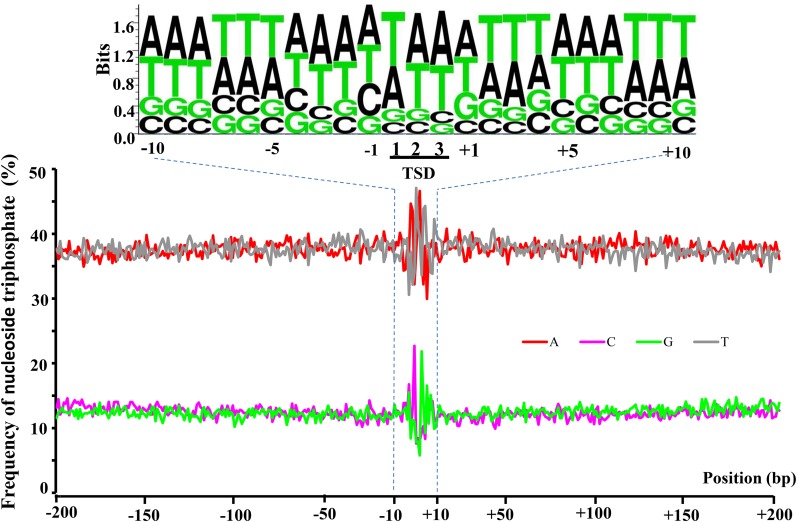
Frequency of nucleoside triphosphate at the 400 bp region surrounding the 3 bp TSD. Positions 1, 2, and 3 represent the 3 bp TSD, positions from −200 to −1 represent the 200 bp upstream region, and positions 1–200 show the 200 bp downstream of the TSD. The 3 bp TSD and 20 bp flanking regions are marked by blue dashed lines. TSD, target site duplications.

Although pvCACTA1 transposons were widely dispersed in the G19833 genome, their insertions were not random as higher transposon densities were found at chromosomal termini. To determine if the pvCACTA1 transposons exhibited insertion site biases, we extracted the 3 bp TSDs and 400 bp flanking sequences (200 bp for each side) of the 2523 complete pvCACTA1 elements and determined the base counts for each position. The GC contents ranged from 13.9–33.7% and with an average GC content of 24.7% across the 403 bp positions. However, the average GC percentage for the 3 bp TSD positions were 15.4%, 16.3%, and 13.9%, named positions 1, 2, and 3, much lower than all other flanking positions (Figure 5). The genome-wide GC content was 35.0% for G19833 and 35.3% for BAT93, much higher than the 24.7% average GC content in the flanking regions and the 15.2% in the TSDs. This indicates that the pvCACTA1 transposons had strong insertion preferences, and preferentially inserted into AT-rich regions and generated AT-rich 3 bp TSDs (WWW, where W is A or T).

### Genic pvCACTA1 transposons

To find genes containing pvCACTA1 transposon sequences, the six complete pvCACTA1 transposons (Figure 1) were used to search against the annotated gene sequences in G19833 (Schmutz *et al.* 2014). A total of 579 genes were found that had pvCACTA1 sequences including 76 genes containing complete elements. Some genes contained more than two copies of pvCACTA1. For example, a 6580 bp gene, *Phvul.008G245400* encoding an NBS-LRR disease resistance protein, contained two complete pvCACTA1 elements in its first and second introns (Figure S2). The vast majority of genic pvCACTA1 transposons were in introns, but we did find 11 expressed genes in which pvCACTA1 sequences had been recruited as exons, including as coding sequences (CDSs) and untranslated regions (UTRs) (Table S1). Among these 11 genes in G19833, 10 contained pvCACTA1 in their CDSs and/or 3′UTRs, but only one had a pvCACTA1 transposon in the 5′UTR. Based on BLAST, nine of the 11 genes had homologous genes in soybean and other genomes. It is interesting to note that these nine genes had ratios of nonsynonymous substitutions per nonsynonymous site (Ka) to the number of synonymous substitutions per synonymous site (Ks) less than 1.0 (Table S1), suggesting that these genes have undergone purifying selection. The pvCACTA1 sequences recruited as exons in these nine genes were shared by the two common bean genomes, G19833 and BAT93, thus these pvCACTA1 sequences are likely fixed within common bean.

To define gene ontologies (GO) for the pvCACTA1-related genes, the 579 gene sequences were used to query the *Arabidopsis thaliana* GOSlim database (ftp://ftp.arabidopsis.org/home/tair/Ontologies/Gene_Ontology). 557 genes could be assigned GO classification, the other 22 genes either shared no sequence identity with proteins in *Arabidopsis* or their homologous proteins had no GO assignment in the database. GO summary based on the molecular function category indicated that 159 genes were related to binding, while 237 genes may be involved in catalytic activity including hydrolase activity (70 genes), transferase activity (63 genes), and kinase activity (15 genes). Additionally, 17 genes likely play roles in gene regulation as all were assigned to the GO category transcription regulator activity (Figure S3A). The most abundant groups under biological process category were linked to metabolic processes (173 genes) and cellular processes (120 genes) (Figure 3B). Of interest, there were 56 genes annotated as response to stimuli, which suggests that these genes may be associated with adaptation to the environment.

### Polymorphisms of pvCACTA1 between the two common bean genepools

We compared the distribution of pvCACTA1 elements between the two sequenced genomes to assess insertion/excision after the divergence of the two gene pools, estimated at ∼0.11 MYA (Mamidi *et al.* 2013). As recent active transposons are usually intact (Jiang *et al.* 2003), we inspected only complete pvCACTA1 transposons and their flanking sequences (100 bp on each side). Since DNA transposons move from one genomic location to another by a cut-and-paste mechanism, precise excisions would restore the insertion site to its original sequence, confounding analysis (Thibault *et al.* 1999; van de Lagemaat *et al.* 2005; Gao 2012). Therefore, we used polymorphic elements to represent the pvCACTA1 transposons that have likely been inserted in or excised from the genomes after the radiation. We used the 2523 complete pvCACTA1 elements with their flaking sequences in G19833 to search against the BAT93 genome. Ninety pvCACTA1 elements were present only in G19833, likely inserted into G19833 or excised from BAT93 after their divergence. The other elements were either not shared by the two genomes, in repetitive sequences, or the flanking sequences were not found. We also used 2150 complete pvCACTA1 transposons and flanking regions from the BAT93 genome to screen the G19833, of which 63 elements were absent in G19833. The polymorphic transposon density was an average of 0.19 polymorphic elements/Mb in G19833 (90/472.5 Mb) and 0.14 polymorphic elements/Mb in BAT93 (63/458.2 Mb), where 472.5 Mb and 458.2 Mb represented the genome sizes of G19833 and BAT93 after the removal of gaps. However, some regions showed higher polymorphic densities. For example, we analyzed a 650 kb subtelomeric region in BAT93, which contains disease resistance gene clusters (David *et al.* 2009) and compared it to the homologous region in G19833. We identified two complete and one nearly complete (30 bp deletion in the 5′TIR) pvCACTA1 elements in BAT93, and one complete pvCACTA1 in G19833, which were polymorphic between the two genomes (validated by PCR, Figure S4).

Further investigation revealed that most of the polymorphic pvCACTA1 transposons were located in intergenic regions. However, 10 polymorphic pvCACTA1 elements were found in genes and may play a role in the divergence of gene sequences. Nine of the polymorphic transposons were located in introns, whereas one element was found in the third exon of the *Phvul.007G248500*, a gene encoding gibberellin 3-β-dioxygenase 4-like protein (Table 1).

**Table 1:** Ten genes with polymorphic pvCACTA1 transposons between accessions G19833 and BAT93

Genome	Genomic Location	Targeted Gene ID	Encoded Protein	Location in Gene
G19833	Chr01 (6234097-6234463)	Phvul.001G054400	Pleiotropic drug resistance ABC transporter family protein	23rd intron
G19833	Chr04 (3321678-3322059)	Phvul.004G030300	Polypyrimidine tract-binding protein	1st intron
G19833	Chr04 (5580231-5580612)	Phvul.004G048000	Disease resistance protein	3rd intron
G19833	Chr05 (27664796-27665197)	Phvul.005G093100	Homeobox-leucine zipper protein	3rd intron
G19833	Chr08 (6575058-6575430)	Phvul.008G072100	Rpp4C3 resistance protein	3rd intron
BAT83	Scaffold00021 (10475-10878)	Phvul.011G189700	Protein zinc induced facilitator-like	11th intron
BAT83	Scaffold00070 (1019591-1019972)	Phvul.006G129200	Peroxidase	2nd intron
BAT83	Scaffold00613 (190211-190535)	Phvul.007G248500	Gibberellin 3-β-dioxygenase 4-like	3rd exon
BAT83	Scaffold00719 (168127-168512)	Phvul.007G112700	Mitogen-activated protein kinase	1st intron
BAT83	Scaffold00775 (68320-68712)	Phvul.011G211800	MATE efflux family protein FRD3-like	8th intron

### Evolutionary dynamics of pvCACTA1 transposons

To explore the evolutionary relationships among the pvCACTA1 transposons, all complete pvCACTA1 elements in both G19833 and BAT93 were used to conduct phylogenetic analyses. After removing divergent elements and ambiguously aligned positions from pvCACTA1 alignments, a phylogenetic tree was built with MEGA (Tamura *et al.* 2007). The pvCACTA1 elements were grouped into nine clades, and elements from both genomes were mixed in all clades except clade V (Figure S5), indicating that these pvCACTA1 elements likely existed before the radiation of the two gene pools. It is interesting that all pvCACTA1 elements in clade V were derived only from G19833, and the vast majority of the elements in clade VII were from BAT93. This suggests that there were amplifications of pvCACTA1 transposons subsequent to the divergence of the two gene pools. To provide more insight into the evolution and diversification of pvCACTA1 transposons, we further classified the complete elements into different subfamilies using a criterion of ≥ 85% sequence identity and ≥ 85% coverage of the elements. From this, the 2523 complete pvCACTA1 elements in G19833 were grouped into 844 subfamilies. The most abundant four subfamilies, named G-SF1 to G-SF4 (G-SFs indicate subfamilies of pvCACTA1 in G19833), contained 582, 176, 124, and 118 complete elements, respectively, for a total of 39.6% of the complete elements in G19833. There were 702 subfamilies containing only one element each; the other 138 subfamilies contained 2–45 elements. In the BAT93 genome, 2150 complete transposons were divided into 895 subfamilies; the top two subfamilies, B-SF1 and B-SF2, contained 509 and 356 elements that accounted for 40.2% of the complete transposons in the BAT93, respectively. The third to 135th subfamily contained 2–32 pvCACTA1 elements, and the other 760 subfamilies contained single elements.

**Figure 6:**
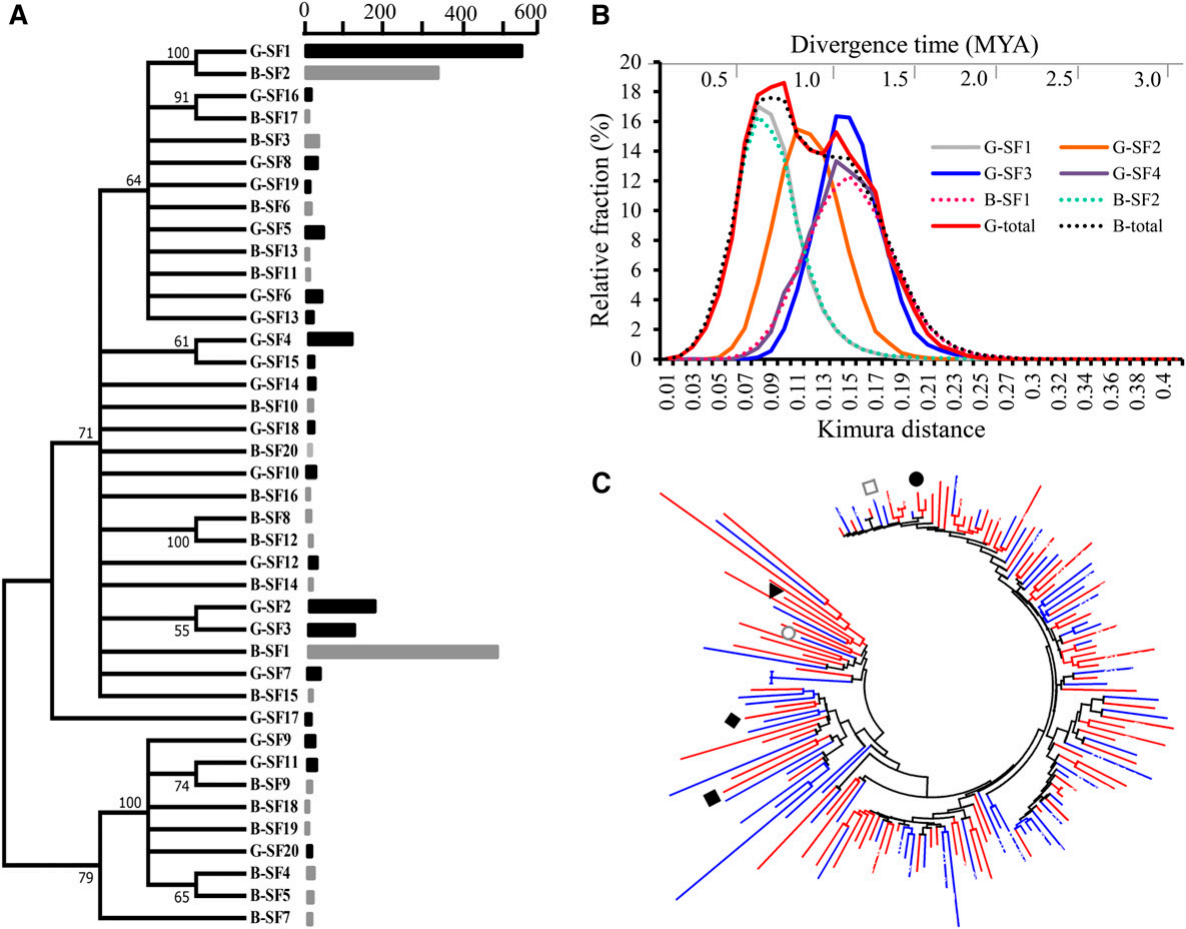
(A) A phylogenetic tree of 20 subfamilies of pvCACTA1 transposons in G19833 and BAT93. The black and gray blocks represent the copy number of each pvCACTA1 subfamily in G19833 and BAT93. (B) The sequence divergence of pvCACTA1 transposons. (C) The phylogenetic tree of the polymorphic pvCACTA1 transposons. The red and blue branches indicate the polymorphic elements in G19833 and BAT93, respectively. The representative elements for the G-SF1 to G-SF4 subfamilies are marked by black circle, square, triangle, and rhombus, respectively. The representative element of the B-SF1 and B-SF2 subfamilies are indicated by a gray circle and a square. MYA, million years ago.

To reduce the complexity of analyses, we made a phylogenetic tree using representative elements of the top 20 subfamilies from each genome. The top 20 subfamilies covered 1342 and 1050 complete elements in G19833 and BAT93, or 53.2% and 48.8% of the total complete elements, in the two genomes, respectively. The element of the first subfamily in G19833 was grouped with the second subfamily from BAT93, and the first subfamily in BAT93 was grouped closely with the second and third subfamilies from G19833 (Figure 6A). To investigate sequence divergence of pvCACTA1 elements, we carried out an all-by-all pairwise sequence divergence analysis and calculated the genetic distance among all alignments using the Kimura two-parameter model (Tamura *et al.* 2007). To avoid a biased assessment, we did this for only six subfamilies (G-SF1 to G-SF4, B-SF1, and B-SF2), as other subfamilies contained a small number of complete elements ranging from 1–45. The distributions of pairwise divergences varied for the six subfamilies (Figure 6B). The G-SF1 and B-SF2 subfamilies exhibited similar divergence distribution patterns, with a divergence peak at 0.08 substitutions/per site. The divergence profile of B-SF1 was different from G-SF1 and B-SF2 as it has a lower and broader divergence peak, which indicates that the B-SF1 subfamily may be an older subfamily. Additionally, the divergence rates of the B-SF1 family were significantly greater than both G-SF1 and B-SF2 (T-test, *P* = 0) and the rates between G-SF1 and B-SF2 subfamilies show no statistical significance (T-test, *P* = 0.64). However, the distribution pattern of B-SF1 was similar to that of G-SF3 and G-SF4, and with a peak at 0.14–0.15 substitutions/per site (Figure 6B).

The pvCACTA1 elements are recently identified transposons and their mutation rate has not been studied. To determine the divergence times and evolutionary dynamics of pvCACTA1 elements, we first estimated the average substitution rate for pvCACTA1 transposons. We manually inspected the genomes of G19833 and BAT93 and extracted 100 pairs of orthologous complete elements shared by the two common bean genomes. The divergence rate (K value) for each pair of orthologous transposon was estimated using the Kimura two-parameter model; the average divergence rate of the pvCACTA1 family was 0.0146 substitutions/per site. We next calculated the average substitution rate (r) of pvCACTA1 transposons with the formula T = K/2r, where K is the average divergence rate of 0.0146 and T is the divergence time of the two genomes (T = 0.11 MY, Mamidi *et al.* 2013). From this, the average substitution rate of the pvCACTA1 family was 6.63 × 10^−8^ substitutions/synonymous site/year. This average substitution rate was used to convert the divergence rates (Kimura distance) into divergence times, the results of which indicate that massive bursts of transposition occurred in both genomes around 0.7 and 1.1 MYA before the divergence of the two gene pools (Figure 6B).

Since six pvCACTA1 subfamilies in G19833 and BAT93 exhibited different amplification dynamics, and the G-SF1 and B-SF2 subfamilies contained more similar or evolutionary young elements than the other four subfamilies, we hypothesized that the G-SF1 and B-SF2 subfamilies would likely have more polymorphic insertion sites than other subfamilies between the two genomes. To test this hypothesis, we carefully examined each subfamily to see if it contained polymorphic elements. Among 90 polymorphic elements in G19833, 24 and 3 were in the subfamilies G-SF1 and G-SF2, respectively, and only one polymorphic element each for G-SF3 and G-SF4. The ratio of the polymorphic elements to the total elements was 0.041 for the G-SF1 subfamily, higher than for G-SF2 (3/176 = 0.017), G-SF3 (1/124 = 0.008), or G-SF4 (1/118 = 0.008). Among 63 polymorphic transposons in BAT93, 9 and 44 were in the B-SF1 and B-SF2 subfamilies, respectively. The ratio of the polymorphic elements to the total elements was 0.018 (9/509) for B-SF1 and 0.124 (44/356) for B-SF2. These results indicate that the G-SF1 and B-SF2 subfamilies were more polymorphic than the other four subfamilies. We further built a phylogenetic tree with the polymorphic elements from G19833 and BAT93, and most of the elements grouped with the representative elements from the G-SF1 and B-SF2 subfamilies (Figure 6C).

## Discussion

### Unusual features of the new CACTA transposon family

CACTA superfamily transposons have been reported in many plants including legumes such as soybean and *L. japonicus* (McClintock 1954; Vodkin *et al.* 1983; Shirsat 1988; Chopra *et al.* 1999; He *et al.* 2000; Kawasaki and Nitasaka 2004; Holligan *et al.* 2006). However, CACTA transposons have not been described in the *Phaseolus* genus, which last shared common ancestors with soybean and *L. japonicas* 20 and 50 MYA, respectively (Lavin *et al.* 2005). Here, we identified a small CACTA transposon family in the common bean named pvCACTA1 that does not show sequence identity with previously described transposons and, thus, represents a new CACTA transposon. Compared to other transposons, the pvCACTA1 family has some unusual features. First, its TIRs can be more than 150 bp in length, much larger than all previously reported CACTA transposons (10–54 bp) (Kunze and Weil 2002; Feschotte and Pritham 2007). Second, pvCACTA1 transposons were very abundant, present in more than 12,000 copies. To our knowledge, this may be the most abundant CACTA transposon family reported thus far. Third, many CACTA transposons are dispersed in plant genomes and are frequently found in genic regions (Vodkin *et al.* 1983; He *et al.* 2000; Zabala and Vodkin 2008). However, the pvCACTA1 transposons are not uniformly distributed along chromosomes and were enriched in telomeric/subtelomeric regions (Figure 3 and Figure 4). Additionally, pvCACTA1 transposons have strong insertion biases as they were often found in AT-rich regions and generated 3 bp TSD of WWW (W is A or T) (Figure 5), similar to that of Tourist MITEs (Bureau and Wessler 1994; Zhang *et al.* 2004), even though they were likely derived from large transposons from the *PIF/Harbinger* superfamily (Jiang *et al.* 2003).

### Origin and amplification of the pvCACTA1 family

Based on transposon display, pvCACTA1 transposons were detected only in common bean and other *Phaseolus* species, but not in cowpea and soybean (Figure 2). Using pvCACTA1 elements as queries against GenBank, we identified homologous sequences in runner bean and tepary bean. However, no homolog was found in non-*Phaseolus* species, including legumes such as cowpea and soybean. The *Phaseolus* genus last shared common ancestors with cowpea and soybean around 8 and 20 MYA, respectively (Lavin *et al.* 2005; Delgado-Salinas *et al.* 2006). We cannot rule out the possibility that the pvCACTA1 family was present in other legumes and that the elements have either diverged or been eliminated; however, it is unlikely that all homologs were lost in all the genomes. Therefore, we propose that the pvCACTA1 transposons represent a lineage-specific transposon family that likely emerged after the radiation of cowpea and the *Phaseolus* genus. Unlike retrotransposons, DNA transposons move via a cut-and-paste mechanism. Thus, their copy number does may not vary as rapidly. However, copy number can increase via the gap repair mechanism or by insertion during DNA replication (Feschotte and Pritham 2007). It still is not clear how and when the pvCACTA1 transposons amplified to reach such high copy numbers. Our data suggests that the massive amplifications of the pvCACTA1 transposons occurred in the ancestral genome of the two common bean gene pools (Figure 2). The *Phaseolus* genus contains 50 wild and cultivated species, and only two genotypes of cultivated common bean have been sequenced (Schmutz *et al.* 2014; http://mazorka.langebio.cinvestav.mx/phaseolus/); genomic resources for other species are very limited. When other whole-genome sequences from other *Phaseolus* species are available, we will be able to compare the pvCACTA1 distributions across different genomes, which should shed more light on the evolutionary dynamics of these transposons.

We identified 90 and 63 polymorphic pvCACTA1 transposons in the G19833 and BAT93 genomes, respectively, which indicates that transposition events likely occurred after the divergence of the two gene pools, ∼0.11 million years (MYA) (Mamidi *et al.* 2013). Polymorphisms were detected among species and even within a gene pool (Figure 2), which suggests that transposition occurred after the emergence of the *Phaseolus* genus and that some elements were recently inserted or excised. Even though more than 2000 complete pvCACTA1 were identified in both G19833 and BAT93, many subfamilies have likely accumulated mutations and become silent or “dead,” and only a few subfamilies may still be able to be mobilized by an autonomous transposon. There are two lines of evidence for this hypothesis: 1) the vast majority of pvCACTA1 subfamilies were either low copy or single copy; and 2) the G-SF1 and B-SF2 subfamilies contained many more polymorphic elements than did the other subfamilies.

The pvCACTA1 elements are small and nonautonomous, that is they have no coding capacity and their mobility depends on proteins encoded by an autonomous partner. We searched the genome sequences of G19833 and BAT93 with the pvCACTA1 transposons and found no large, protein-encoding element that shared sequence identity. The possible reasons are: 1) the autonomous transposon was either lost or has diverged, but this is unlikely as it is difficult to imagine how an autonomous element dramatically diverged within such a short period of time (0.11 MY); 2) The autonomous element was not captured in the common bean draft genomes as many autonomous transposons were present in single copy or low copy (Jiang *et al.* 2003; Zhang *et al.* 2004; Yang *et al.* 2009); and 3) The autonomous element may not share significant sequence identity with pvCACTA1, *i.e.*, they may have moved by cross-mobilization (Yang *et al.* 2009).

### Function and potential applications of pvCACTA1

Transposons are known to play crucial roles in genome evolution and gene innovation (Wisman *et al.* 1998; Feschotte and Pritham 2007). We analyzed the common bean genome and found 579 genes that contained pvCACTA1 sequences. Of these, 557 showed significant sequence identity with genes in *Arabidopsis* and could be assigned GO classification (Figure S3, A and B). The GO classifications clearly indicated that these genes were involved in a variety of biological processes and important molecular functions such catalytic activity and gene regulation. Most of the pvCACTA1 elements appear to have inserted into genes before the divergence of the two gene pools. For instance, we manually inspected 76 genes containing complete pvCACTA1 elements in the G19833 genome: 71 genes were shared between G19833 and BAT93, only five genes harbored pvCACTA1 elements that were polymorphic between the two genomes (Table 1). Interestingly, we identified nine genes that contained pvCACTA1 sequences in the CDSs and/or UTRs; all were shared between the two gene pools and may have been fixed by artificial or natural selection. Four and one pvCACTA1-related ESTs were found in runner bean and tepary bean, respectively. These results suggest that the pvCACTA1 transposons contributed to gene formation and divergence in common bean and possibly in related *Phaseolus* species. Even though there were elements that had integrated into genes, the majority of the pvCACTA1 transposons were located in intergenic regions, which included 85 and 58 polymorphic complete elements in G19833 and BAT93, respectively. These elements may have a role in gene regulation if located in regulatory regions, play structural roles, or contribute to nonhomologous subtelomeric recombination (Daniel *et al.* 2003), or they may be neutral.

Apart from their impact on gene and genome evolution, transposons have been widely used as an important resource for developing transposon-based markers and gene-tagging systems (Wisman *et al.* 1998; Casa *et al.* 2000; Hancock *et al.* 2011). The pvCACTA1 transposons are abundant in common bean and are found in other *Phaseolus* species; therefore, they may be used to develop new molecular markers for phylogenetic studies or for conducting marker-assisted breeding in *Phaseolus* species.

## Supplementary Material

Supplemental Material
