# Supplementary material for: Dynamics of a Novel Highly Repetitive CACTA Family in Common Bean (Phaseolus vulgaris)
Source: G3 (Bethesda). 2016 May 16;6(7):2091–101. doi: 10.1534/g3.116.028761 (PMC4938662; doi:10.1534/g3.116.028761)
Supplement: Supplemental Material [file supp_6_7_2091__index.html]

Dynamics of a Novel Highly Repetitive CACTA Family in Common Bean (Phaseolus vulgaris) — Supplemental Material 

# Dynamics of a Novel Highly Repetitive CACTA Family in Common Bean (*Phaseolus vulgaris*)

## Supplemental Material for Gao *et al.*, 2016

**Files in this Data Supplement:**

- Table S1 - A summary of 11 genes containing pvCACTA1 sequences in exons. (.pdf, 165 KB)
- Figure S1 - A phylogenetic tree of common bean and other legumes. (.pdf, 144 KB)
- Figure S2 - A gene contains two complete pvCACTA1 transposons at the position of 355-738 and 2663-3042. (.pdf, 24 KB)
- Figure S3 - Summary of the pvCACTA1 transposon related genes using gene ontology terms according to molecular function (A) and biological process (B). (.pdf, 81 KB)
- Figure S4 - A. Comparison of a 650-kb region in BAT93 and its orthologous sequence in G19833. (.pdf, 65 KB)
- Figure S5 - A phylogenetic tree of pvCACTA1 elements from BAT93 (black) and G19833 (red). (.pdf, 362 KB)
