# Supplementary material for: Dynamics of a Novel Highly Repetitive CACTA Family in Common Bean (Phaseolus vulgaris)
Source: G3 (Bethesda). 2016 May 16;6(7):2091–101. doi: 10.1534/g3.116.028761 (PMC4938662; doi:10.1534/g3.116.028761)
Supplement: Supplemental Material [file supp_g3.116.028761_TableS1.pdf]

Table S1. A summary of 11 genes containing pvCACTA1 sequences in exons

Genes in common bean				Homologous gene in soybean	Ka/Ks ratio
Name	mRNA	Location of pvCACTA1	Encoding protein		
Phvul.010G050600	XM_007134416	CDS and 3'UTR	ethylene-responsive transcription factor 2-like	LOC100792115	0.40
Phvul.011G211500	XM_007133757	3'UTR	cellulose synthase A catalytic subunit 3 [UDP-forming]-like	LOC100790276	0.06
Phvul.001G069900	XM_007161388	CDS and 3'UTR	stress-induced receptor-like kinase	LOC100305367	0.15
Phvul.006G211800	XM_007148413	3'UTR	mitogen-activated protein kinase kinase kinase 1-like	LOC100806902	0.19
Phvul.008G001600	XM_007139042	3'UTR	leucine-rich repeat extensin-like protein 6-like	LOC100778270	0.31
Phvul.001G018300	XM_007160745	3'UTR	probable transcription factor KAN4-like	LOC100778242	0.25
Phvul.003G290700	XM_007156432	3'UTR	glucan endo-1,3-beta-glucosidase-like protein 2-like	LOC100779749	0.36
Phvul.003G021000	XM_007153207	3'UTR	protein TIFY 6B-like	LOC100799314	0.53
Phvul.009G228200	XM_007138609	3'UTR	transcription factor MYBZ1	LOC778159	0.45
Phvul.005G018900	XM_007148782	5'UTR	hypothetical protein	NA	
Phvul.001G018200	XM_007160744	3'UTR	hypothetical protein	NA	
