# Supplementary material for: Dynamics of a Novel Highly Repetitive CACTA Family in Common Bean (Phaseolus vulgaris)
Source: G3 (Bethesda). 2016 May 16;6(7):2091–101. doi: 10.1534/g3.116.028761 (PMC4938662; doi:10.1534/g3.116.028761)
Supplement: Supplemental Material [file supp_g3.116.028761_FigureS1.pdf]

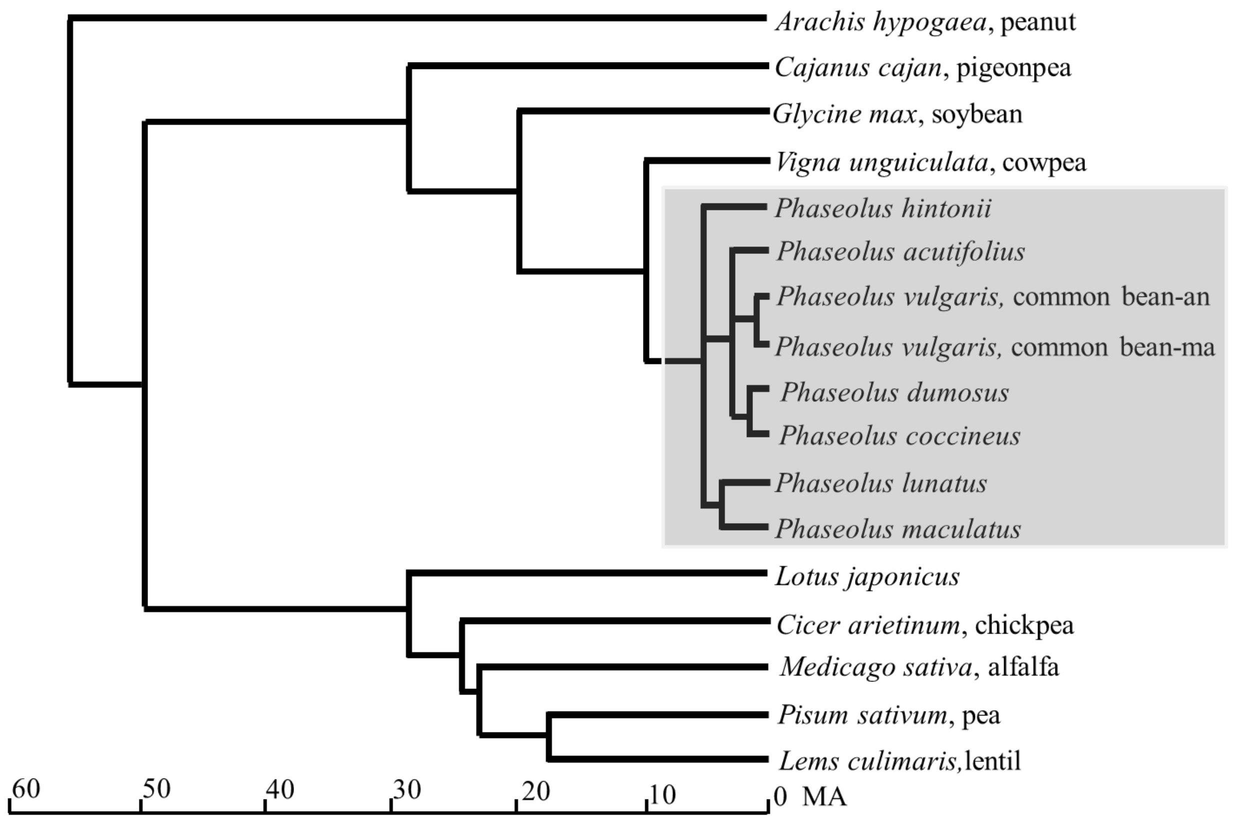

Figure S1. A phylogenetic tree of common bean and other legumes. The tree was modified based on the published results from Lavin et al (2005) and Delgado-Salinas et al (2006). Common bean-an and common bean-am represents the Andean and Mesoamerican gene pools, respectively. The *Phaseolus* species are marked by grey block.
