# Supplementary material for: Dynamics of a Novel Highly Repetitive CACTA Family in Common Bean (Phaseolus vulgaris)
Source: G3 (Bethesda). 2016 May 16;6(7):2091–101. doi: 10.1534/g3.116.028761 (PMC4938662; doi:10.1534/g3.116.028761)
Supplement: Supplemental Material [file supp_g3.116.028761_FigureS2.pdf]

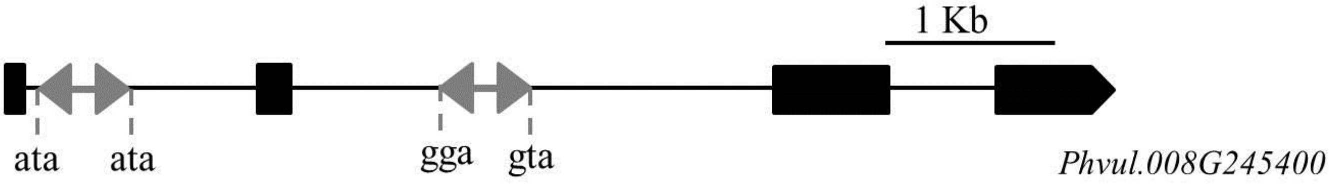

Figure S2. **A gene contains two complete pvCACTA1 transposons at the position of 355-738 and 2663-3042.** The black boxes and lines are the exons and introns of the *Phvul.008G245400* gene. The grey triangles are the TIRs of pvCACTA1 elements.
