# Supplementary material for: Dynamics of a Novel Highly Repetitive CACTA Family in Common Bean (Phaseolus vulgaris)
Source: G3 (Bethesda). 2016 May 16;6(7):2091–101. doi: 10.1534/g3.116.028761 (PMC4938662; doi:10.1534/g3.116.028761)
Supplement: Supplemental Material [file supp_g3.116.028761_FigureS3.pdf]

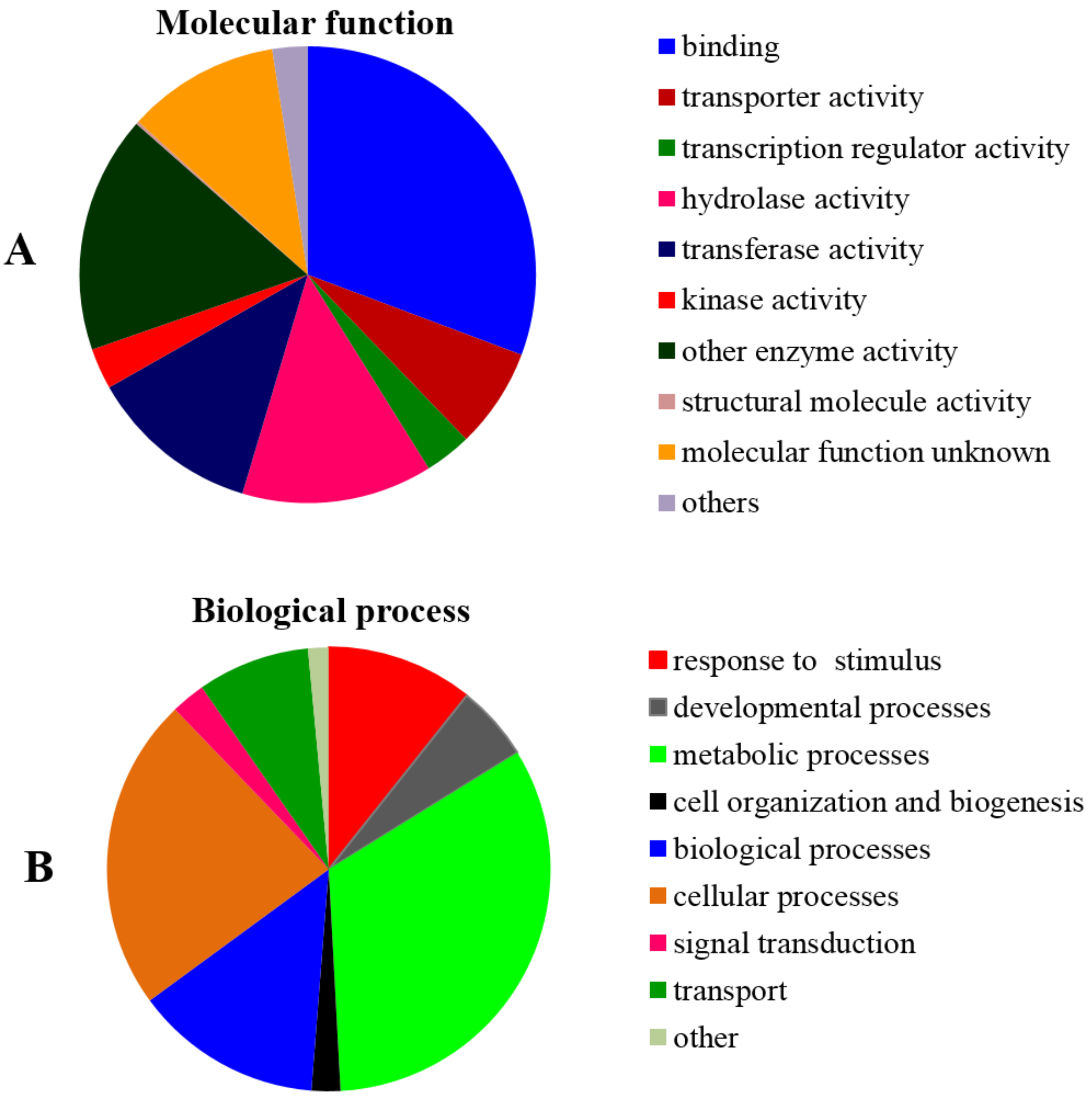

Figure S3. Summary of the pvCACTA1 transposon related genes using gene ontology terms according to molecular function (A) and biological process (B).
