# Supplementary material for: Dynamics of a Novel Highly Repetitive CACTA Family in Common Bean (Phaseolus vulgaris)
Source: G3 (Bethesda). 2016 May 16;6(7):2091–101. doi: 10.1534/g3.116.028761 (PMC4938662; doi:10.1534/g3.116.028761)
Supplement: Supplemental Material [file supp_g3.116.028761_FigureS4.pdf]

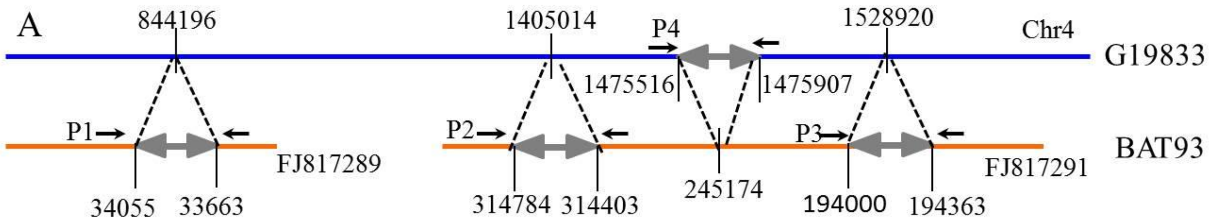

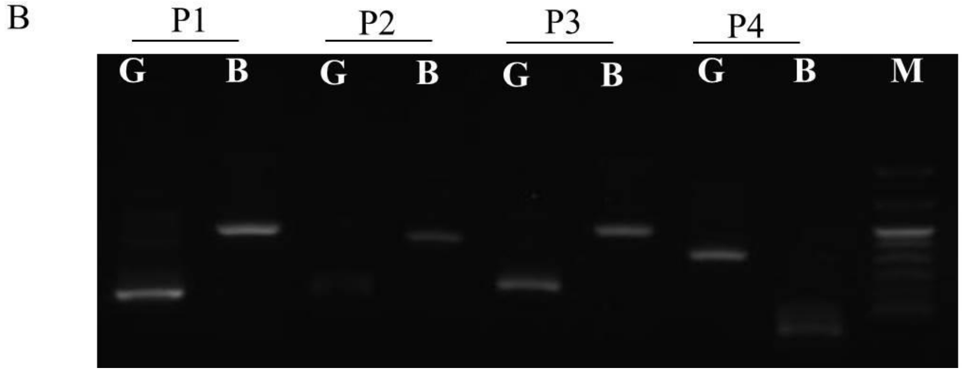

Figure S4. **A. Comparison of a 650-kb region in BAT93 and its orthologous sequence in G19833.** The blue and orange lines represent the genomic sequences of G19833 and Bat93. The grey triangles are the TIRs of pvCACTA1 elements. B. **PCR analysis of four polymorphic pvCACTA1 elements between G19833 and BAT93**. G and B mean the DNA from G19833 and BAT93, respectively. The primers used for PCR analysis are indicated by black arrows in A.
