# Supplementary figures and images for: Dynamics of a Novel Highly Repetitive CACTA Family in Common Bean (Phaseolus vulgaris)
Source: G3 (Bethesda). 2016 May 16;6(7):2091–101. doi: 10.1534/g3.116.028761 (PMC4938662; doi:10.1534/g3.116.028761)

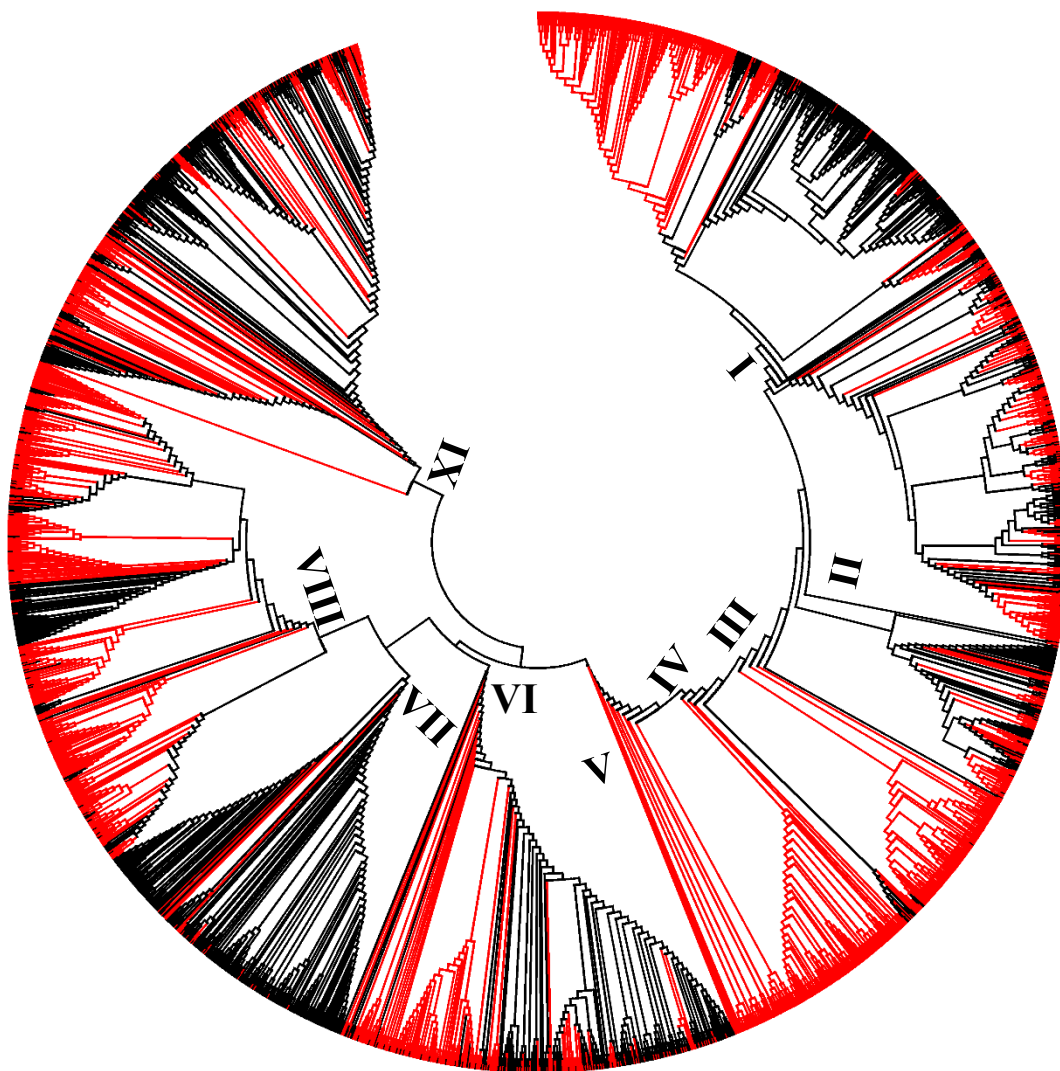

**Figure S5.** A phylogenetic tree of pvCACTA1 elements from BAT93 (black) and G19833 (red).

Supplement: Supplemental Material [file supp_g3.116.028761_FigureS5.pdf]
